# Responsible laboratory surveillance of pediatric patients with inflammatory bowel disease on biologic infusion therapy

**DOI:** 10.1002/jpr3.70154

**Published:** 2026-02-12

**Authors:** Tanmai Shah, Ashwin Agrawal, Héctor E. Alcalá, Anupama Chawla

**Affiliations:** ^1^ Department of Pediatrics Stony Brook Children's Hospital, Renaissance School of Medicine Stony Brook New York USA; ^2^ Division of Pediatric Gastroenterology, Department of Pediatrics Robert Wood Johnson University Medical School New Brunswick New Jersey USA; ^3^ Department of Family, Population and Preventive Medicine Stony Brook University Hospital, Renaissance School of Medicine Stony Brook New York USA; ^4^ Division of Pediatric Gastroenterology and Nutrition, Department of Pediatrics Stony Brook Children's Hospital, Renaissance School of Medicine Stony Brook New York USA

**Keywords:** cost, Crohn's disease, infliximab, management, ulcerative colitis

## Abstract

The annual healthcare spending on pediatric inflammatory bowel disease (IBD) has increased over time. The objectives of the study were to investigate the cost of routine laboratory surveillance in pediatric IBD patients during outpatient maintenance biologic infusions and determine their impact on clinical management. We performed a retrospective chart review between June 30, 2020 and July 1, 2021 of subjects aged 0–21 years with IBD receiving maintenance biologic infusions at Stony Brook Children's Hospital (SBCH). Thirty‐four subjects met the inclusion criteria. A total of 248 lab sets/infusions were reviewed during the study window. The average hospital charge for studies obtained per infusion was $1308.36 ± $136.66 with an average annual cost of $9543.44 ± $1885.44 per patient. Fifteen (6%) instances of change in clinical management were found. Our study suggests routine laboratory surveillance during infusions does not affect clinical management and may represent excess financial spending.

## Abstract (highlights)

**What is Known:**

- In 2016, the estimated total healthcare spending for pediatric patients with Inflammatory Bowel Disease (IBD) was $2 billion making up 8% of the national total healthcare spending.
- Pediatric ambulatory healthcare spending on IBD was estimated to be $836 million in 2016, an 8.1% increase since 1996.
- Routine laboratory surveillance with outpatient biologic infusions is common place.

**What is New:**

- Annual cost of routine laboratory surveillance in Pediatric IBD patients creates a substantial financial burden for families and the healthcare system of $9543.44 ± 657.86 per patient.
- Routine laboratory surveillance seldom results in clinical management changes.

## 1 INTRODUCTION

Inflammatory bowel disease (IBD), including Crohn's disease and ulcerative colitis, are chronic inflammatory pathologies of the gastrointestinal tract. These disorders are often diagnosed in adolescence and young adulthood and have had a rising incidence in pediatric populations.^1^ The incidence of pediatric inflammatory bowel disease in the United States is estimated to be 2.4–8.2/100,000 person‐years.^2^ In 2016, the estimated total healthcare spending in pediatric patients with IBD was $2 billion making up 8% of the nationally estimated total healthcare spending on IBD. Unlike in adult populations, the majority of healthcare spending in pediatric patients is done in an ambulatory setting, followed by emergency and inpatient settings respectively. Pediatric ambulatory healthcare spending on IBD was estimated to be $836 million in 2016, an 8.1% increase since 1996^3^, ^4^ of which infusion services make up a major portion.

Once IBD has been diagnosed, the main goals of therapy include achieving remission, improving quality of life, and limiting adverse effects of therapy.^1^, ^5^ With the emergence of monoclonal antibodies such as infliximab and vedolizumab, the treatment of pediatric IBD has shifted from immunosuppressants to biologic infusions. With this transition, therapeutic drug monitoring (TDM) has become more common place in pediatric IBD treatment regimens. Studies have demonstrated the utility of TDM in improving clinical outcomes, however, optimal frequency has yet to be defined.^6^, ^7^, ^8^ Other screening laboratory studies, such as those used to assess for anemia and nutritional status, also do not have a defined optimal frequency. Frequent laboratory studies may be placing an unnecessary financial burden on the healthcare system as it is unclear the extent to which they affect clinical management.

The primary objective of this retrospective study is to identify the costs of laboratory studies obtained during maintenance biologic infusion visits. The secondary objective of this study is to assess how frequently routine laboratory studies result in changes in clinical management.

## 2 METHODS

### 2.1 Ethics statement

The study was conducted at Stony Brook Children's Hospital and was approved by Stony Brook University institutional review board with the designation IRB2021‐00531.

### 2.2 Study population

A retrospective chart review of pediatric patients with IBD biologic infusion therapy at Stony Brook Children's Hospital (SBCH) was performed. Pediatric patients with IBD were identified by an internal list of patients receiving hospital‐based infusion therapy. The inclusion criteria was: (1) be between the ages of 0 and 21 years old, (2) receiving maintenance biologic infusion therapy between June 30, 2020 and July 1, 2021, and (3) receive infusion therapy at SBCH exclusively during the entire study period. Patients with indeterminate IBD were included in the study if the above criteria were met.

### 2.3 Data capture

Each subject's data was de‐identified and entered into a specifically created SBUH REDCap database, a university hospital affiliated electronic data capture tool.

### 2.4 Data collection

The following data points were collected: IBD diagnosis (Crohn's disease vs. ulcerative colitis vs. indeterminate IBD), infusion medication (Infliximab vs. Inflixmab biosimilar vs. Vedolizumab), number of infusion sessions during study window, documented pediatric disease activity index scores (pediatric Crohn's disease activity index [PCDAI] or pediatric ulcerative colitis activity index [PUCAI]), type and frequency of routine laboratory studies obtained during study window, and changes in clinical management due to the laboratory studies.

Routine laboratory studies were defined as those part of the standardized infusion protocol at SBCH and were obtained with each scheduled infusion. The following laboratory studies were considered routine/standard: complete blood count with differential, basic metabolic panel, liver function tests, amylase, lipase, erythrocyte sedimentation rate, C‐reactive protein, vitamin D, iron, ferritin, vitamin B12, folate, urine hCG (if a subject was female). Other laboratory studies that were collected, but not considered routine studies included QuantiFERON‐TB, and biologic drug and antibody level.

Changes in clinical management due to laboratory studies were obtained by reviewing documented ambulatory patient progress notes. Criteria for changes in clinical management were defined prior to data collection. Criteria were defined as follows: (1) change in biologic infusion dosage, (2) change in biologic infusion frequency, (3) addition of new medication as part of IBD therapy, (4) addition of new medication to treat nutritional deficiency, (5) cessation of medication, (6) repeating laboratory testing at a shorter interval, (7) performance of an endoscopic procedure, (8) performance of a radiographic study, and (9) abnormal laboratory study result requiring referral to the emergency department or inpatient admission.

### 2.5 Charge determination

Hospital charges for each laboratory study included were obtained from the SBUH billing department (Table 1). Hospital charge is defined as the charge SBUH applies to a patient or insurance company for a specific service.

**Table 1:** Laboratory study charges.

Laboratory study	Charge (Dollars)
Complete blood count with differential	$136
Basic metabolic panel	$226
Liver function test	$299
Amylase	$144
Lipase	$150
Erythrocyte sedimentation rate	$86
C‐reactive protein	$22
Vitamin B	$179
Vitamin D	$488
Iron	$132
Ferritin	$195
Folate	$196
Biologic drug and antibody level	$539
Urine hCG	$271
QuantiFERON‐TB Gold	$365

## 3 RESULTS

### 3.1 Sample characteristics

There were 34 patients identified who met criteria for inclusion in the study. Of the 34 patients, 23 (68%) had a diagnosis of Crohn's disease, 11 (32%) had a diagnosis of ulcerative colitis, and 0 patients had indeterminate IBD (Table 2). The average number of infusions during the study window was not found to be significantly different (*p* = 0.287) between populations with 7.4 and 7.1 infusions for patients with Crohn's disease and ulcerative colitis respectively.

**Table 2:** Patient sample characteristics.

	Variable	Quantity or mean (%)
Diagnosis	Crohn's disease	23 (68%)
	Ulcerative colitis	11 (32%)
	Indeterminate inflammatory bowel disease	0 (0%)
Biologic use in patient sample	Infliximab	26 (76%)
	Infliximab biosimilar	2 (6%)
	Vedolizumab	6 (18%)
Biologic use in Crohn's disease patients	Infliximab	23 (100%)
	Infliximab biosimilar	0 (0%)
	Vedolizumab	0 (0%)
Biologic use in ulcerative colitis patients	Infliximab	3 (27%)
	Infliximab biosimilar	2 (18%)
	Vedolizumab	6 (55%)
Average Disease Activity Index Score	PCDAI	4.5
	PUCAI	3.5
Average number of infusions	Crohn's disease	7.4
	Ulcerative colitis	7.1

Abbreviations: PCDAI, Pediatric Crohn's Disease Activity Index; PUCAI, Pediatric Ulcerative Colitis Activity Index.

Disease activity index scores were averaged within their respective populations. As each disease activity index follows a different scale PUCAI scores were multiplied by a conversion factor of 1.176 to achieve a 0–100 scale, as used by PCDAI, to better optimize data analysis. The average disease activity index scores were not found to be significantly different (*p* = 0.816) between the two groups with a score of 4.5 for patients with Crohn's disease and a score of 3.5 (4.01 after conversion) for patients with ulcerative colitis.

### 3.2 Laboratory surveillance cost

The average annual charge per patient, charge per infusion, and total annual charge were calculated using the hospital charge and frequency of laboratory studies ordered within the study window. The cost for the biologic medication and the infusion visit was not included. The charge for biologic drug levels was not included in the charge calculations as drug levels were typically obtained at the discretion of the provider and not routinely with each infusion. However, these tests were included when reviewing for changes in clinical management.

The total annual charge amongst all patients in the study was $324,447 (Table 3). There was no statistically significant difference (*p* = 0.668) between the average annual charge per patient between patients with Crohn's disease and Ulcerative colitis. The average annual laboratory charge per patient amongst all patients in the study was $9543.44 ± $657.86 with an average laboratory charge per infusion of $1308.36 ± $47.68 (Table 3).

**Table 3:** Laboratory surveillance charges.

	Total annual charge	Average annual charge per patient	Average charge per infusion
Crohn's disease	$221,756.00	$9641.57 ± 860.27	$1319.96 ± 57.90
Ulcerative colitis	$102,721.00	$9,338.27 ± 1155.01	$1320.64 ± 100.10
All patients	$324,447.00	$9,543.44 ± 657.86	$1308.36 ± 136.66

### 3.3 Impact on clinical management

248 infusions and their respective laboratory studies were performed within the study window. Of the 248 infusions, clinical management changes due to laboratory studies were found in only 15 instances (6%) (Table 4). Changes consisted of change in infusion frequency or biologic dosage, addition of new medication as part of IBD therapy or to treat a nutritional deficiency, cessation of medication, and repeat laboratory testing at a shorter interval. No instances of endoscopic procedure performance, radiographic studies, or emergency department referrals were found. Of the 15 laboratory studies selected from the standardized infusion protocol, only three (Vitamin D level, iron level, and biologic level) were found to have led to the changes in clinical management.

**Table 4:** Changes in clinical management secondary to laboratory studies.

Change	Quantity	Percentage of total laboratory study sets
Change in infusion frequency	4	1.6%
Change in biologic dosage	4	1.6%
Addition of new medication as part of inflammatory bowel disease therapy	1	0.4%
Addition of new medication to treat nutritional deficiency	2	0.8%
Cessation of medication	2	0.8%
Repeat laboratory testing at shorter interval	2	0.8%
Performance of endoscopic procedure	0	0%
Performance of radiographic study	0	0%
Referral to emergency department	0	0%
Total clinical changes	15	6%

## 4 DISCUSSION

The results suggest that routine laboratory studies at the time of infusion infrequently lead to changes in clinical management. As only 6% of instances of laboratory studies led to changes in management, excess surveillance may be an unproductive financial burden. On closer review, only a limited subset of the 15 laboratory studies included were utilized in making changes: biologic drug, Vitamin D, and iron levels. These changes were minor and could have been effectively addressed with less frequent testing. A first step towards responsible laboratory surveillance could involve decreasing the frequency at which these studies are obtained. This could be achieved by staggering laboratory studies throughout maintenance based on their clinical utilization. Studies with less clinical utility may be performed less frequently compared to studies with higher utility. An alternative method of limiting spending may be stratifying surveillance based on disease severity. Our study population had well controlled disease as evident by low PCDAI and PUCAI scores, and found to have few changes in management likely due to the patients all being in the maintenance phase of therapy. This population may require less frequent surveillance compared to patients with higher disease activity. Future studies focusing on laboratory surveillance in patient populations with higher disease activity would allow for better understanding of the utility of stratification of surveillance based on disease activity.

Our study was conducted in 2020–2021. Prior to this, there were no guidelines published or recommendations regarding frequency of laboratory surveillance to our knowledge. In 2023 the American Gastroenterological Association (AGA) reported conditional recommendations for laboratory surveillance every 6–12 months for adult patients in symptomatic remission with UC. However, it does not differentiate between patients on biologic therapy versus other therapies.^9^ In addition, the AGA reported for adult patients in symptomatic remission of Crohn's disease, conditional recommendation with low certainty of evidence for checking interval biomarkers every 6–12 months.^10^ In 2024, Improve Care Now recommended all patients receive periodic laboratory surveillance at least every 6–12 months or more frequently if indicated.^11^ Our study helps provide more evidence that frequency of laboratory monitoring in patients with IBD can be reduced in the appropriate patient and setting.

The study has a few important limitations. As a retrospective study, data collection and analysis are limited by information documented from prior visits. Insufficient or inaccurate documentation could impact data collection and analysis. While patients on maintenance therapy may benefit from less frequent laboratory surveillance, patients on induction or newly diagnosed may not necessarily benefit in the same way. Third, we acknowledge that our study cohort was limited to 34 patients in clinical remission. A larger cohort would be needed to verify the generalizability of the frequency of test results can be reduced without harm. We believe our cohort size was appropriate to generalize the cost of laboratory studies, which was the primary objective. Additional studies including patients with higher disease activity indexes are needed to see if less frequent lab monitoring would be appropriate in that setting. Fourth, our study focused on hospital‐based laboratory surveillance and did not include patients who received laboratory services at outside facilities. The costs of these laboratory tests were not factored in our analysis but imply a greater annual cost than what was found. Lastly, the study used the hospital charge set by our institution's billing department to quantify cost. This value can vary among institutions and third‐party laboratory services and does not reflect the amount paid by the patient nor their insurance. Future studies utilizing these reimbursement amounts for laboratory surveillance or utilizing insurance database studies could quantify healthcare expenditure more accurately.

## 5 CONCLUSION

Our observations suggest the utility of routine laboratory surveillance at each biologic infusion is minimal, favoring decreased testing for IBD patients, especially those in clinical remission. Utilizing disease activity index scores may serve as an effective way to stratify patients and reduce laboratory surveillance. We propose obtaining laboratory tests twice a year, or with every third infusion, for patients with mild disease or in remission based on their disease activity index scores. In our small cohort of patients, this change in practice would reduce the total annual costs by 66% ($214,154.82). Our study suggests observing mindfulness when ordering labs in IBD patients receiving infusions would aid in reducing healthcare expenditure.

## CONFLICT OF INTEREST STATEMENT

The authors declare no conflict of interest.
